# Supplementary material for: Are carbon emissions trading and green financial instruments synergistic? -Comprehensive quantitative research based on content analysis
Source: PLoS One. 2024 Mar 7;19(3):e0298601. doi: 10.1371/journal.pone.0298601 (PMC10919601; doi:10.1371/journal.pone.0298601)
Supplement: S1 Data — (ZIP) [file pone.0298601.s001.zip › Data/地级市Z_F工作报告.zip!/╔╜╢1⁄2/╤╠╠¿╩╨/└┤╫╘╣1⁄2╓┌║┼í╛┬φ┐╦╩2╛▌═°í┐_2003.docx]

一、开拓创新的五年

“1998年至2002年的五年，是全市人民坚持邓小平理论和‘三个代表’重要思想，不断开拓创新的五年。”这是代市长周齐在市第十四届人民代表大会第一次全体会议上作政府工作报告时，对五年来全市各项事业的发展给予的充分肯定。

他说，五年来，全市上下在上级党委、政府和中共烟台市委的正确领导下，坚持以经济建设为中心，解放思想，与时俱进，团结拼搏，扎实工作，较好地完成了市十三届人大一次会议以来确定的各项任务，国民经济和社会各项事业取得了新的成就。

———综合经济实力跃上新的台阶。2002年，国内生产总值预计可达1115亿元，是1997年的1.75倍，年均递增11.8％；金融机构各项存款余额达到1179亿元，是1997年的1.8倍，年均递增12％；全社会固定资产投资完成419亿元，是1997年的2.9倍，年均递增24％；境内财政总收入完成107.4亿元，其中地方财政收入完成45.2亿元，是1997年的2.6倍，年均递增21.1％，继续保持收支平衡。

———经济结构进一步优化。一、二、三次产业增加值分别达到136亿元、574.5亿元和404.5亿元，三次产业结构比例由1997年的18∶51.3∶30.7调整为12.2∶51.5∶36.3。农业内部结构优化升级，农业外向度由41％提高到56％，全市规模以上工业完成销售收入1229亿元，实现利税117亿元，分别是1997年的1.9倍和2.2倍，年均分别递增13.2％和16.8％。第三产业对国民经济的贡献率明显提高。

———各项改革纵深推进。以完善法人治理结构、建立现代企业制度为目标的国企改革进一步深化，全市企业改制面达到97.8％。国有资产管理体制改革迈出较大步伐，在市级企业中初步建立起了国有资本出资人制度。个体私营经济快速发展，非公有制经济规模迅速扩大，占全市企业资产总额的比重增加到53.9％。市场融资成果显著，五年中新增5支上市股票。在全省率先建立了市级行政审批中心。市场导向的就业机制基本形成。农村税费改革圆满完成，农民人均减负25.8％。

———对外开放成效显著。全市五年累计实际利用外资32.3亿美元，相当于开放以来利用外资总额的50.7％。去年全市实现外贸进出口总额44.8亿美元，外贸依存度达到33.2％，其中出口25.7亿美元，是1997年的1.9倍，年均递增13％。烟台经济技术开发区、APEC科技工业园、出口加工区等各类园区发展迅猛。

———基础设施和城市建设力度加大。全市五年累计完成城建投资150亿元，交通建设投资120亿元，进一步完善了城市基础设施和市政公用设施，提升了城市整体形象。

———各项社会事业全面发展。全市五年共取得科技成果994项，其中获国家、省科技进步奖280项。全市城市居民人均可支配收入8869元，农民人均纯收入3826元，城乡居民人均储蓄11900元，分别比1997年增加2954元、974元和4490元。

———精神文明建设和民主法制建设日益加强。深入开展了多种形式的群众性精神文明创建活动。文化、科技、医疗“三下乡”活动深入推进。争创全国双拥模范城活动成效显著。村民自治制度在农村普遍推行。同“法轮功”邪教组织的斗争取得决定性胜利。廉政建设和反腐败斗争向纵深发展，查处了一批大案要案，维护了公务员队伍和政府机关的形象。

二、开启现代化建设新篇章

周齐在政府工作报告中强调，从现在到2007年的五年，是我市改革开放和现代化建设承前启后的关键时期，是全面建设小康社会、实现跨越式发展的重要阶段，是必须紧紧抓住并且可以大有作为的重要战略机遇期。我们要审时度势，站在时代高度谋划烟台未来，努力开启现代化建设的新篇章。

周齐提出，未来五年政府工作总的任务是：全面贯彻党的十六大精神，高举邓小平理论伟大旗帜，认真实践“三个代表”重要思想，围绕全面建设小康社会、率先基本实现现代化，以加快发展为第一要务，以结构调整为主线，以改革开放和科技进步为动力，进一步解放思想，开拓创新，加快国际化、市场化和城市化进程，大力实施科教兴烟战略和可持续发展战略，提高经济发展活力和竞争能力，推动经济社会跨越式发展，不断提高人民生活水平，为把烟台建成区域性中心城市而努力奋斗。

周齐强调，今后五年，我市要努力实现六个跨越。一是，加快经济结构战略性调整，提高国民经济整体素质，奋力实现综合实力的新跨越。到2007年，力争国内生产总值和地方财政收入比2002年翻一番，分别突破2000亿元和100亿元。发展速度确保高于全省2－3个百分点，高于全国14个首批沿海开放城市的平均水平。积极扩大投资规模，增强发展后劲，力争五年累计完成全社会固定资产投资3500亿元。大幅度调整优化农业内部结构，大幅度提高高新技术产业比重，大幅度提高服务业比重。一、二、三次产业结构调整为8∶51∶41,高新技术产业比重超过30％。二是，充分发挥区位优势，加快建设具有较强辐射带动力的对外开放基地，奋力实现经济国际化的新跨越。要始终坚持以对外开放统揽全局，全面推进各个领域的对外开放。特别要紧紧抓住日、韩产业结构调整的重要机遇，加速建设具有国际竞争力的出口加工制造基地。全市五年累计实际利用外资力争突破100亿美元，比上一个五年增长2倍以上；2007年外贸出口突破50亿美元，力争比2002年翻一番，外贸依存度提高到40％。积极实施“走出去”战略，力争在境外投资、劳务输出等领域实现大的突破。三是，打破城乡二元结构，加快建设现代化港口城市和滨海生态旅游胜地，奋力实现城市化的新跨越。坚持城乡统筹，突出城市在发展中的主导作用。面向本世纪中叶，构建以中心城市、中等城市、小城市和卫星镇为发展极，以骨干路网和滨海旅游线为主体框架，布局合理、功能互补的城乡蓝图。起步建设牟平至莱州沿海一线，串连各个港口、各类园区，绵延200公里的产业隆起带和滨海生态旅游线。充分发挥港口优势，整合交通和物流资源，发展临港产业。加速膨胀以五区为主体的中心城市规模，放开户籍限制，强化产业支撑，大力吸纳转化农业人口，提高县级市驻地和15个中心镇的人口聚集度。力争到2007年，全市有一半以上人口居住在城镇，城市化水平达到50％以上。四是，大力推进市场取向的各项改革，充分激发多种经济成份的发展活力，奋力实现市场化的新跨越。以放开、放宽、放活为主要手段，大力发展民营经济，大力推进体制创新、制度创新和环境创新，加速完善统一、开放、竞争、有序的现代市场体系。基本完成所有制结构和政府管理模式的战略性调整，非公有制经济占全市工商企业资产总额的比重达到80％以上，政府管理经济的运作机制基本实现法制化。五是，以富民为第一目标，加快全面建设小康社会步伐，奋力实现人民生活质量的新跨越。到2007年，力争跨上人均国内生产总值4000美元的大台阶。城市居民人均可支配收入达到15000元以上，人均住房使用面积达到18平方米；农民人均纯收入达到5000元以上，人均住房使用面积达到32平方米。劳动力平均受教育年限由2002年的9.5年提高到12年。国民健康、生态环境和社会信息化水平进入全国前列。同时，要加大扶贫力度，关注弱势群体，努力增加就业，提高社会保障水平。六是，全面发展先进文化和各项社会事业，弘扬奋发向上的精神风貌，奋力实现向现代文明城市的新跨越。要把与时俱进、开拓创新，作为推动烟台跨越式发展的不竭动力，永不满足，追求卓越。大力倡导崇尚学习、尊重人才的社会风尚，大力强化社会公德、职业道德和家庭美德教育，大力弘扬友爱互助、乐于奉献的集体主义精神，不断提高民主与法制建设水平，广泛开展多种形式的群众性精神文明创建活动，营造祥和安定的社会环境，努力使市民素质和城市形象在全面建设小康社会进程中得到新的升华。

三、2003年工作重点

高点定位开好局

2003年是贯彻党的十六大精神、全面建设小康社会的第一年，也是新一届政府工作的起步年。周齐在政府工作报告中要求各级、各部门解放思想，高点定位，扎实做好今年的工作，为实现未来五年的奋斗目标开好头。

周齐建议今年全市经济和社会发展主要预期目标安排为：国内生产总值增长14％，地方财政收入增长18％，全社会固定资产投资增长20％，社会消费品零售总额增长12％；外贸出口增长16％，实际利用外资增长25％；城市居民人均可支配收入增长12.5％，农民人均纯收入增长6％；城镇登记失业率控制在3.5％以内，人口自然增长率控制在3.3‰以下。

在更高层次上参与国际竞争

周齐在政府工作报告中指出，要以招商引资和扩大出口为重点，在更大范围、更广领域和更高层次上参与国际经济技术合作和竞争，推进外贸主体和出口市场多元化，实现引资区域多元化。

周齐强调，要进一步加大招商力度，创新招商方式，拓展招商领域，积极寻求与国内外大公司建立战略性合作关系，探索采用风险投资、股权转让、海外上市等方式利用外资。大力吸引日、韩资金，加大欧美国家的引资力度。要积极扶持出口导向型企业，加快出口产品结构调整，高度重视进口工作，实现“以进带出”。要大力实施“走出去”战略，广泛开展国际合作，同时，进一步完善服务体系，提高效率，优化环境，开创参与国际经济技术合作和竞争新局面。

加快推进新型工业化进程

周齐在政府工作报告中提出，以传统产业高新化和高新技术产业化为重点，加快推进新型工业化进程。

周齐指出，要大力培植支柱行业，通过增加投入建设一批新项目、引进技术改造一批老企业，为机械制造、纺织服装、食品加工、黄金四大传统行业注入新的活力，使电子信息、新型材料、生物制药等新兴行业加快膨胀。要大力培植骨干企业，继续实施“2358工程”，加大对张裕、万华、南山、丛林等骨干企业和上海通用东岳汽车、浪潮LG、泰信电子等新兴企业的引导扶持力度，促其加快体制创新、机制创新、技术创新。要大力发展高新技术产业，重点支持万华、氨纶等10户高新技术企业，集中力量扶持高性能永磁材料、YH－16等10个高新技术生长点；优先发展信息产业，加快烟台IT工业园建设，实施制造业信息化示范工程，以信息化带动工业化。要大力推进企业技术改造，加快用高新技术和先进适用技术改造提升传统产业步伐，搞好产品延伸，提高产品档次，提升技术、工艺、装备水平和自主创新能力。

纵深推进农业产业化

周齐在政府工作报告中指出，农业和农村工作是政府工作的重要部分，要以纵深推进农业产业化经营为重点，开辟农民增收和农村经济发展的新路子，加快农村现代化建设步伐。

周齐强调，要继续调整农业结构，调减一般粮田面积，扩大优质经济作物种植面积，发展壮大新兴产业。加大绿化造林工作力度，改善生态环境。要加快发展农村专业合作经济组织，提高农民的组织化程度。扩大名优海珍品养殖，推进“海上烟台”二次创业。周齐指出，要积极培育农业知名品牌，提高市场竞争力，促进更多的农产品进城出境。要加强农业基础设施建设，改善农业生产条件，提高农业机械化水平。坚持工农结合，城乡统筹，多渠道增加农民收入，争取用三年的时间，从第一产业转移出劳动力50万人，由农民转市民50万人。

全面提高服务业素质

周齐在政府工作报告中强调，要以发展商贸物流、旅游会展和金融业为重点，全面提高服务业整体素质和竞争能力。

周齐说，要围绕推进流通现代化，大力发展连锁经营、物流配送和现代市场，不断完善城市社区商业服务网络，扩大农村连锁商业网点。要充分发挥交通特别是口岸优势，理顺港口管理体制，提高吞吐能力。要抓好物流信息平台、物流园区、配载配送中心等关键设施建设，加快发展第三方物流。要突出旅游特色，整合旅游资源，建设滨海生态旅游长廊。要进一步搞好旅游产品促销，促进旅游与商贸、会展、文化、体育等产业的有机结合。要以创建金融安全区为目标，不断扩大融资和信贷规模。同时要积极争取国内外金融企业来烟设立分支机构，加快发展证券、保险和担保业，进一步搞活资本市场。

加快所有制结构调整

周齐在政府工作报告中要求，各级政府部门要以大力发展民营经济为重点，加快所有制结构调整步伐。

周齐强调，要进一步强化民本经济意识，毫不动摇地鼓励、支持、引导非公有制经济特别是个体私营经济发展。全面落实各项扶持政策，放宽民营资本的市场准入领域，努力营造全民创业的机制、环境和氛围。要支持个体私营企业以多种形式参与国有、集体企业改制、改组和改造，引导个体私营企业加快管理和技术创新，建立规范的法人治理结构。要深化国有资产管理体制改革，进一步完善国有资本出资人制度。大力推行规范的公司制改革，完善法人治理结构。要进一步深化县域企业特别是镇村企业的产权制度改革，积极探索矿山等资源性企业的改革和公益事业单位的改革。年内，市县乡村四级企业产权制度改革比重达到75％，完成38户市属企业改制任务，核销呆坏帐2.5亿元以上。

千方百计增加有效投入

周齐在政府工作报告中强调，要以园区和大项目建设为重点，千方百计增加有效投入，促进全市经济不断登上新台阶。

周齐指出，要把各类园区建成融资的主要载体和资金投入的密集地。要尽快完成牟平养马岛到蓬莱栾家口港，再到莱州三山岛的整合开发，使沿海产业隆起带建设取得新突破。在大项目建设方面，要完成渤海天然气供气项目主管网和二级管网建设，加快206高速、烟台至蓬莱滨海旅游观光路、烟大铁路轮渡、黄烟铁路建设步伐，争取蓬莱19－3油气田陆上基地落户烟台，积极推进IGCC、龙口电厂调峰机组和胶东供水项目，继续争取海阳核电、核能海水淡化项目。

周齐进一步指出，要把利用外资作为增加有效投入的关键措施，面向国内外大公司、大集团，积极寻求合作，引进大项目、大资金。要采取多种形式搞好项目推介活动，主动为企业和银行牵线搭桥，争取金融支持。要进一步拓宽投资准入领域，最大限度地挖掘民间资本的投资潜力。年内，力争完成全社会固定资产投资500亿元。

加快城市化进程

周齐在政府工作报告中强调，城市化是我市政治、经济、社会发展的必然趋势和要求，全市上下要围绕“年年有变化，三年见成效，五年大变样”的目标，以膨胀中心城市、搞活县域经济为重点，加快城市化进程。

周齐指出，要以规划为龙头，进一步搞好城市规划的集中统一管理，全面推行规划设计方案招标制，加大规划执法力度。要以加快基础设施建设为突破口，继续抓好重点路段、重点工程建设，搞好老城区的综合整治。要进一步理顺城市管理体制，加大综合行政执法力度，提高城市管理水平。要进一步做好经营城市的文章，严格执行各项制度，高度垄断土地一级市场，努力增加土地收益。周齐强调，对县域经济，要加强分类指导，引导各县市区高点定位，突出特色，发挥优势，增强产业竞争力。要加快城镇化步伐，突出抓好县级市驻地和强镇建设，提高规划水平和建设品位，增强承载力和吸引力。同时，继续加强对落后县市的帮扶工作，促进县域经济协调发展，全面推进城市化进程。

集中办好十件实事

周齐在政府工作报告中要求政府部门重点围绕群众最关心、最急需解决的热点难点问题，集中办好十件实事。

这十件实事的主要内容是：旧城区及居民小区综合整治，市区路网及管线建设与改造，市区绿地建设和主干道两侧绿化美化，新建经济适用房和廉租房，实施放心菜篮子工程，完善和新建老年福利设施，搞好困难群体再就业援助和基层劳动保障网络建设，深入开展爱心救助工作，优化配置教育资源，完善提高城乡社区服务功能和水平。

大力实施科教兴烟战略

周齐在政府工作报告中指出，要以实施科教兴烟战略为重点，繁荣发展各项社会事业。

他说，要大力强化“科学技术是第一生产力、人才是第一资源”的观念，把科技兴市、人才强市贯穿于各项工作之中。要深化科技体制改革，加大科技投入，全面提高科技创新能力。积极引进国内外研发机构，加强企业技术中心建设，抓好各类科技创业园区。大力实施人才战略，加强优秀人才的培养和引进工作，建立紧缺人才培训、引进机制。优先发展教育事业，积极支持驻烟高等院校发展，搞好产学研结合和科技成果转化。合理配置教育资源，鼓励社会力量办学。合理开发利用资源，搞好环境保护，创建生态城市。大力发展文化艺术、新闻出版、广播电视等各项社会事业。

全力维护社会稳定

周齐在政府工作报告中指出，要以维护社会稳定为重点，推进精神文明和民主法制建设。

周齐强调，要进一步加强社会治安综合治理，抓好安全生产，完善处置突发事件的快速反应机制。高度重视信访工作，认真做好失业职工和其他有关方面人员的政治思想工作，有效化解人民内部矛盾。要把推进精神文明建设、提高公民道德素质，作为维护社会稳定的基础工程来抓。要进一步发扬社会主义民主，完善公开办事的各项制度，保证人民群众依法行使民主权利。加强社会主义法制建设，搞好法制宣传教育，提高全民法律素质。

四、建设高效廉洁务实为民的人民政府

“全面建设小康社会，实现我市的跨越式发展，对政府工作提出了新的更高的要求。”周齐在政府工作报告中指出，面临新形势新任务新挑战，各级政府及各部门必须用时代发展的要求审视政府工作，以改革的精神加强自身建设，并自觉运用十六大精神指导工作实践，不断推动工作的创新。

周齐指出，要坚持与时俱进，弘扬创新精神。要把学习、宣传、贯彻十六大精神作为政府工作的首要任务，牢牢把握解放思想、实事求是、与时俱进的思想路线，培养创新作风，健全创新机制，努力在“新思路、新突破、新局面、新举措”上下功夫。要适应新形势新任务的要求，大胆探索，积极实践一切符合“三个有利于”的新做法、新经验。对所有制结构调整中公与私的问题，深化改革中进与退的问题，扩大开放中内与外的问题等，都要以全新的理念去认识、去把握，最大限度地激发和保护广大干部群众的积极性、创造力，不断开拓实践的新境界，努力开创工作的新局面；要坚持依法行政，加快职能转变。按照社会主义市场经济体制的要求，强化各级政府的经济调节、市场监管、社会管理和公共服务职能。以提速增效为目标，深化行政审批制度改革，规范行政行为，改进管理方式，提高服务质量。要进一步完善了解民情、反映民意、集中民智、珍惜民力的决策机制，建立重大事项社会公示和听证制度，完善专家咨询制度，实行决策论证制和责任制，大力推进行政决策的科学化、民主化。要贯彻依法治国方略，严格按照法律规定办事，按照法定程序办事，按照国际规则办事，推行政务公开，切实提高依法行政水平；要坚持求真务实，转变工作作风。坚决反对官僚主义、形式主义，切实防止和克服虚报浮夸、敷衍塞责、推诿扯皮等不良风气。要扎扎实实为基层服务，为民解忧；要坚持行政监察和从严治政，不断加大治本力度，从源头上预防和解决腐败问题，确保各项政令处处畅通和政府各项工作不断提高到一个新水平。
